# When temporal attention interacts with expectation

**DOI:** 10.1038/s41598-024-55399-6

**Published:** 2024-02-26

**Authors:** Aysun Duyar, Shiyang Ren, Marisa Carrasco

**Affiliations:** 1 Department of Psychology, New York University, New York, NY USA; 2 Center for Neural Science, New York University, New York, NY USA

**Keywords:** Psychology, Human behaviour, Cognitive neuroscience, Sensory processing

## Abstract

Temporal attention is voluntarily deployed at specific moments, whereas temporal expectation is deployed according to timing probabilities. When the target appears at an expected moment in a sequence, temporal attention improves performance at the attended moments, but the timing and the precision of the attentional window remain unknown. Here we independently and concurrently manipulated temporal attention–via behavioral relevance–and temporal expectation–via session-wise precision and trial-wise hazard rate–to investigate whether and how these mechanisms interact to improve perception. Our results reveal that temporal attention interacts with temporal expectation–the higher the precision, the stronger the attention benefit, but surprisingly this benefit decreased with delayed onset despite the increasing probability of stimulus appearance. When attention was suboptimally deployed to earlier than expected moments, it could not be reoriented to a later time point. These findings provide evidence that temporal attention and temporal expectation are different mechanisms, and highlight their interplay in optimizing visual performance.

## Introduction

The visual environment is constantly changing, and our brain receives more sensory information than it can process over time due to its limited temporal capacity^1^. Temporal attention and expectation are two key cognitive mechanisms that provide shortcuts to optimize perception. Endogenous temporal attention is the voluntary prioritization of task relevant time, whereas temporal expectation refers to the probability of event timing^2–5^.

Temporal expectations, which are based on the predictability of event timing, increase visual accuracy and decrease reaction times^6,7^. Expectations are developed through the statistical regularities of external events embedded within noisy distributions, resulting in uncertainty regarding event timing. Uncertainty, as a mathematical term, is measured by the variance of a probability distribution^8^. Temporal uncertainty, which is inversely related to the precision of the expectation at a given moment, impairs the performance in visual, auditory, and tactile modalities^9–12^. Another source of temporal expectation is hazard rate: the increasing probability over time of an event occurring, given that it has not yet occurred within a certain time window, facilitates online updating of expectation^4,13–15^.

Temporal attention enables us to select and prioritize specific moments when relevant events occur and enhances the processing of the sensory information at such moments^15–19^. These enhancements in visual perception, which go beyond mere expectation, result in impairments at the unattended moments^3,20^. Hence, temporal attention allocation results in tradeoffs–benefits at the attended moment and concurrent costs at unattended moments. Importantly, there are open questions regarding the timing and the precision of the temporal attentional window: (1) Do people use expectation to guide attention to a stimulus specific point in time or do they merely attend to its order in a sequence of events?; 2) Do the effects of temporal attention vary as a function of the precision with which people attend to the stimulus?

It has been proposed that expectations can guide temporal attention^21,22^. Research suggests that temporal attention is directed towards a behaviorally relevant target when it is expected. When the target has not appeared at the expected moment, observers may shift their attention to the next possible time frame when an event may occur. However, temporal attention and expectation have been used interchangeably or when defined as different mechanisms, they have not been independently manipulated^16,18,23–26^. Thus, a more parsimonious explanation is that hazard rate–the updated expectation with increasing probability of an event occurring, given that it has not yet occurred–can account for these performance differences without necessarily invoking attention allocation. To investigate these alternative hypotheses, attention and expectation should be independently manipulated, to enable the comparison of the effects of attention at expected and unexpected time points.

Attention and expectation both improve performance but modulate neural responses in an opposite manner. In the visual system, temporal attention enhances neural responses to stimuli^25,27^, whereas temporal expectations suppress these responses^28^. In the auditory system, they interact at the neural level, expectation suppresses MEG beta-band oscillations for unattended- but not for attended- stimuli^29^. Unfortunately, in that study the potential perceptual interactions could not be examined because performance was at ceiling. In any case, in the visual system, it is unknown whether attention and expectation interact at the neural or behavioral level.

**Figure 1:**
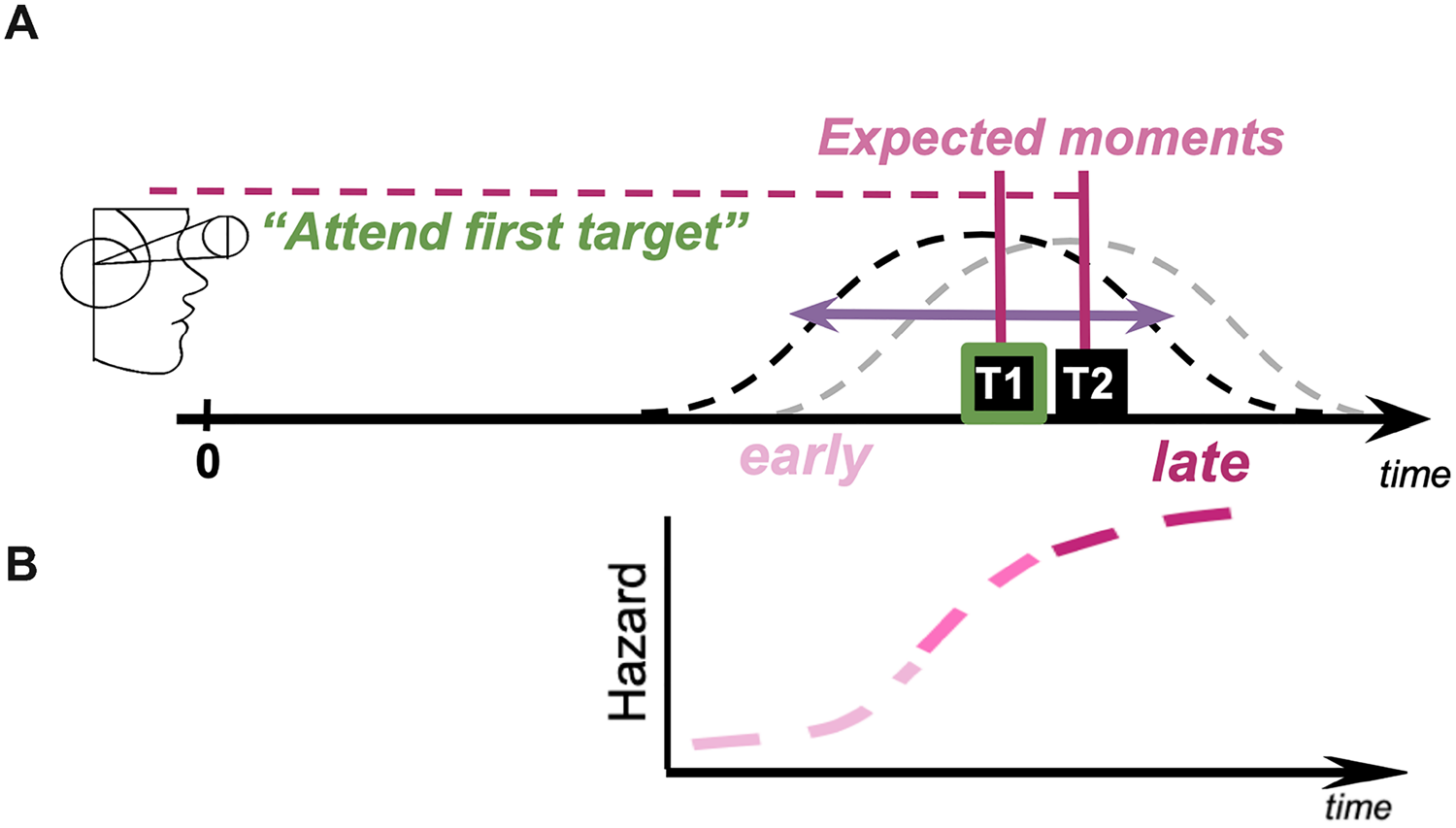
Illustration depicts experimental manipulations of temporal attention, hazard rate and precision. The brain’s temporal processing capacity is limited, and it is a challenge to process the two brief sequential targets perfectly well. (**A**) Temporal Attention is prioritization of a behaviorally relevant moment, and is allocated based on the instructions to selectively process a target that briefly appears (first target, T1, depicted here as an example), and ignore the subsequent one (T2). Given an event with a temporal probability distribution (distribution of possible visual event onsets), we can characterize time relative to the most expected time point: “early”, “expected”, and “late” time points. Temporal Precision describes the inverse of the variability of temporal distribution (shown with a purple horizontal arrow overlaid on T1 temporal probability distribution). (**B**) Hazard Function is a function of time that characterizes the conditional probability of target appearance at every time point, which increases as time passes and the target onset is delayed. The figure illustrates the shape of the hazard function in the current study (arbitrary time, does not represent actual experimental manipulations).

Here, we investigated the benefits of voluntary temporal attention on behavior while manipulating temporal expectation via precision and hazard rate (Fig. 1). We implemented a protocol by combining temporal attention^3,20^ and expectation^29,30^ manipulations. In our protocol, temporal attention–prioritization of a behaviorally relevant moment—is allocated based on the instructions to selectively process a target from two competing stimuli; temporal expectation–ability to guide behavior based on event probability—is manipulated via temporal precision, such that when event timing is certain precision is high, and hazard rate (used to index stimulus onsets earlier than, at or later than the expected moment in low temporal precision sessions) such that within the trial, the probability of stimulus appearance at a given moment increased as the stimulus onset was delayed. This protocol also enables us to distinguish whether people selectively attend to a stimulus at a specific point in time, or alternatively they merely rely on its order in a sequence, regardless of timing or expectations. Our results reveal that both temporal precision and hazard rate influence temporal attention, albeit in different manners.

## Methods

### Observers

Sixteen observers (10 females, six males, aged 22–34 years), including one author (A.D.) have participated in the experiment. The number of observers needed was determined by a power analysis using G*Power software^31^. The effect size was set to *η*_*G*_^2^ = 0.14, which was reported in a previous paper with the same experimental protocol, and comparable number of manipulations^32^, and the sample size was evaluated at 80% power for the potential interaction between temporal attention and expectation.

All observers provided written informed consent and had normal or corrected- to-normal vision. All experimental procedures were in agreement with the Helsinki declaration and approved by the New York University Institutional Review Board.

### Apparatus

Stimuli were generated using an Apple iMac (3.06 GHz, Intel Core 2 Duo) and MATLAB 2012b (Mathworks, Natick, MA, USA) along with the Psychophysics Toolbox (Brainard, 1997; Kleiner et al., 2007), and presented on a CRT monitor that was color-calibrated (1280 × 960 screen resolution, 100-Hertz refresh rate). Observers were seated 57 cm from the display with their head movements limited by a chinrest. The Eyelink 1000 eye tracker (SR Research, Ottawa, Ontario, Canada) was employed to record eye position and perform online eye tracking to maintain central fixation throughout the trials. If a fixation break due to a blink occurred or if the eye position deviated more than 1° from the center of the screen between the ready cue and the response cue, the trial would be aborted and subsequently repeated at the end of each experimental block. Observers could blink or move eyes after the response cue and during the intertrial interval.

### Stimuli

Stimuli were displayed on a uniform medium gray background. A fixation circle (subtending 0.15° dva) was presented at the center of the screen. The placeholders were four small black circles (0.2°) placed at corners of an imaginary square (side length = 2.2°) centered at the screen center.

Target stimuli were Gaussian-windowed (standard deviation of 0.3°) sinusoidal gratings (spatial frequency = 4 cpd) with random phase presented at full contrast. Each target was tilted clockwise (CW) or counterclockwise (CCW) from the vertical or horizontal axis. The tilt was titrated for each observer and for each target interval independently.

Auditory stimuli were presented through the speakers. The attentional precue was a 200-ms auditory tone, either a sinusoidal wave, or a complex waveform that is a combination of sinusoidal waves with frequencies ranging from 50 to 400 Hz. A high-frequency sinusoidal tone (800 Hz) indicated the first target (T1), a low-frequency sinusoidal tone (440 Hz) indicated the second target (T2), and the complex tone was uninformative regarding the target (neutral precue). The physical properties of the auditory tones were selected after the piloting process to ensure that they were easily distinguishable from each other and easily conveyed the attentional instruction to the participants. The same tones used in T1 or T2 were used as the response cue at the end of the trial to instruct the observer to report the orientation of the first (T1) or the second (T2) target, respectively.

### Experimental procedure

The experimental protocol was adapted from previous endogenous temporal attention literature^3,20,32^ and expectation literature^29^. In a two alternative forced-choice task, observers were asked to report the orientation of one of the two Gabor stimuli. Throughout the experiment, the two targets were presented sequentially at the center of the screen, while the observers were fixating at that location.

**Figure 2:**
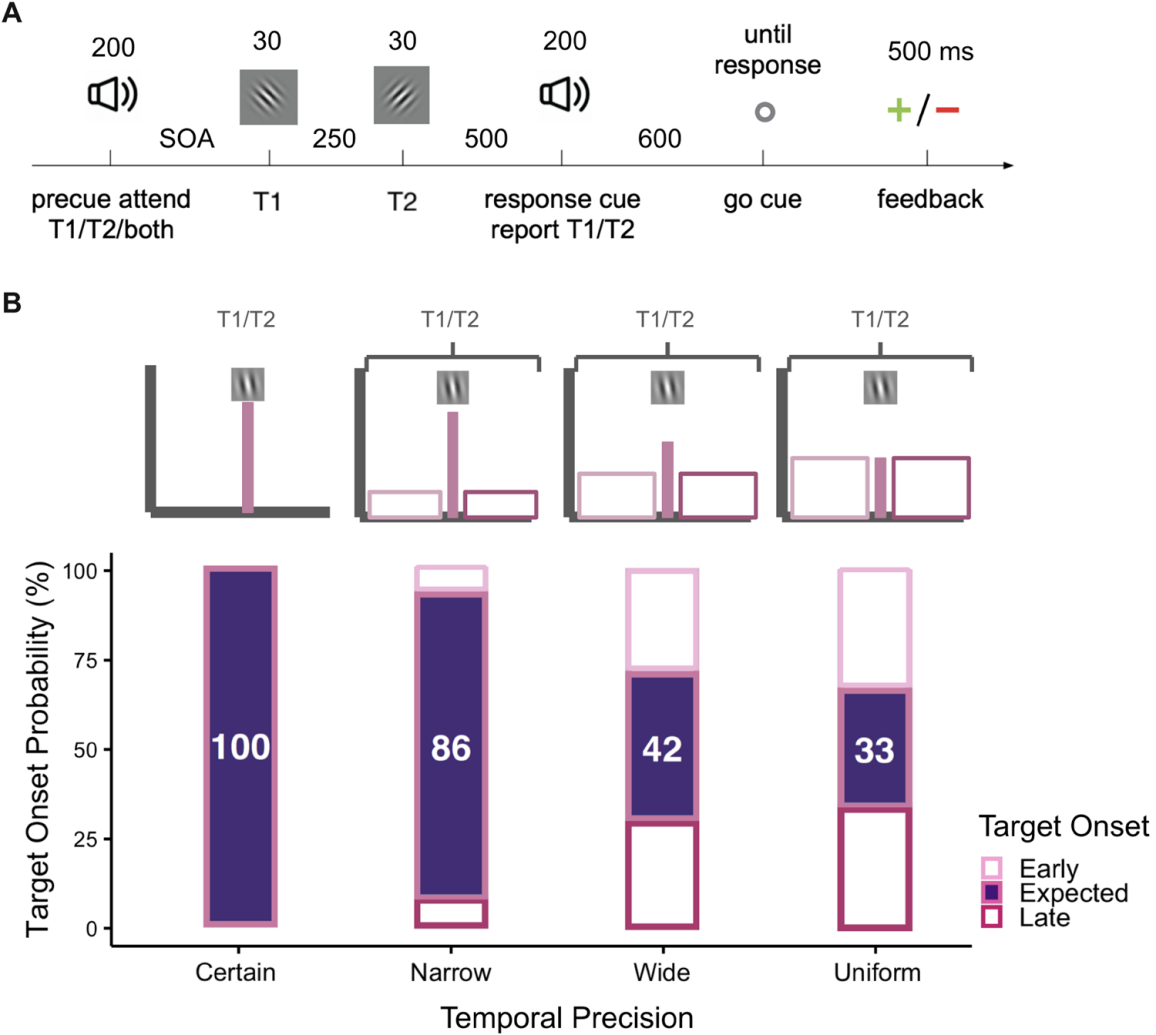
(**A**) Psychophysical procedure (adapted from Denison et al., 2017 to test visual performance at the attended and unattended time points when the stimulus onset is variable. Precue was either neutral, or indicated which target’s orientation will be asked to report at the end of the trial (response cue). Observers had unlimited time to respond, and received feedback based on the accuracy of the response. (**B**) Temporal precision of the stimulus onset was manipulated across sessions, and ranged from certain (no variability, highest precision) to uniform (lowest precision) (adapted from Todorovic et al., 2015)). Values inside the purple bars represent the percentage of trials that the stimuli appeared at the expected moment. For the certain condition, the targets appeared at the expected moment in 100% of the trials. With decreasing precision, targets could also appear earlier or later than this expected time point. Percentage of expected trials dropped with decreasing precision, 86% of the narrow, 42% for the wide and 33% for the uniform conditions.

Figure 2A illustrates the experimental protocol. At the beginning of each trial, an auditory precue was presented to indicate whether to attend to the first target (T1), second target (T2) or both targets. Each target was presented for 30 ms with a stimulus onset asynchrony (SOA) of 250 ms. The onset of the targets relative to the precue was variable throughout the experiment, and was controlled throughout each session to manipulate temporal precision. An auditory 200-ms response cue was presented 500 ms after the second target onset. In valid trials, the precue indicated which target to attend (either T1 or T2), and the response cue was the same tone as the precue, instructing the observer to report the orientation of the cued target. In the neutral trials, the precue was uninformative regarding which target would be task-relevant, and the response cue instructed to report the orientation of either target with 50% probability. Therefore the observers would be selectively attending to the precued target in the valid trials, whereas attending to both targets equally in the neutral trials. The observers were allowed to respond after the go cue, when the fixation brightness changed 800 ms after the response cue onset. We included the go cue in our protocol to encourage observers to prioritize accuracy over speedy responses (as used in temporal attention^3,4,17,32^ and spatial attention protocols^33–35^). Feedback was presented for 500 ms after each trial (A red minus after an incorrect response, or a green plus after a correct response). The observers had no time limit to respond, and were instructed to prioritize accuracy over speed when responding. The intertrial interval was jittered between 1700 and 1100 ms. Eye-tracking was used throughout the experiment, except during the practice session. Participants were instructed to avoid making eye movements and to focus on the center of the screen. If a participant looked away from the center of the screen or blinked during a trial, the trial was skipped and presented again at the end of the block. However, participants were free to make eye movements and blink after the response cue and before the pre-cue of the subsequent trial, defined as their response window.

Temporal precision within an experimental session was manipulated by changing the variability of the stimulus onsets relative to the precue, adapted from a previous neuroimaging study investigating the interaction between temporal expectation and temporal attention^29,30^. Figure 2B illustrates the temporal distributions that created uncertainty–different levels of temporal precision. There were four temporal precision conditions in our design: certain, narrow, wide, and uniform. We tested visual performance at similar levels of stimulus probability as previous studies (33% and 86%)^29,30^, and added two additional temporal precision levels (42% and 100%). In all conditions, the probability distributions of T1 and T2 onsets were centered at 1400 and 1650 ms respectively after the precue onset (expected moments). And the probability of T1 appearing at 1400 ms, and T2 at 1650 ms after the precue onset was either 100% (certain), 86% (narrow), 42% (wide), and 33% (uniform, lowest precision) respectively, which determined the level of temporal precision. For each precision condition, we collected data for 1, 2, 3, or 4 sessions for certain, narrow, wide and uniform conditions respectively, to be able to have enough data points at the mean point (expected moment) of the temporal distribution. The temporal distributions were explained to the observers prior to each experimental session.

Except for the 100% certain condition, there was a time window (1200–1600 ms for T1, and 1450–1850 ms for T2) when the stimuli could occur, which enabled us to test the performance at earlier and later than the expected moment (midpoint of the temporal distributions, 1400 ms for T1 and 1650 ms for T2). In the wide (42%) and uniform (33%) conditions, we had enough trials at the early and late time points that were comparable to the number of trials at the expected moment. Probability of stimulus appearance in the early or late time windows were always equal in all temporal precision conditions. The early and late time windows for both stimuli encompassed 150 ms, and stimulus onset within this 150 ms was randomly selected from a uniform distribution. To ensure that the temporal variability within the early and late windows were equal within each block, we splitted these windows into two smaller 75-ms bins, and ensured an equal number of trials would be in the first and second bins of these early and late temporal windows. The early window was 1200–1350 ms for T1 (equal number of onset for T1_Early_ ∈ [1200, 1275] and T1_Early_ ∈ [1275, 1350]) and 1450–1600 ms for T2 (equal number of onset for T2_Early_ ∈ [1450, 1575] and T2_Early_ ∈ [1575, 1600]), and the late window was 1500–1650 ms for T1 (equal number of onset for T1_Late_ ∈ [1500, 1575] and T1_Late_ ∈ [1575, 1650]) and 1700–1850 ms for T2 (equal number of onset for T2_Late_ ∈ [1700, 1775] and T2_Late_ ∈ [1775, 1850]), such that there was a gap around the expected moment for both targets, in which the targets would not appear (T1 ∉ (1350,1400) ∪ (1400,1450) and T2 ∉ (1600,1650) ∪ (1650,1700)).

The order of the trials was randomized across the sessions, and the order of the sessions for temporal precision was randomized across participants. Each observer completed 7–10 sessions and 3568–4256 trials in total. The experimental sessions with different temporal precision were conducted on separate days (typically, 1–7 days apart). Observers were explicitly shown the corresponding temporal precision at the beginning of each session. Before each session, we titrated the neutral performance at the expected time points (1400 ms for T1 and 1650 ms for T2 after the precue) to be at 75% for both targets independently. Each experimental session started with the tilt threshold determined by the best PEST procedure (Lieberman and Pentland, 1982), and was adjusted on a block-by-block basis if the neutral accuracy significantly differed from the aimed baseline of 75% accuracy.

In summary, we manipulated temporal attention, and two forms of temporal expectation within the same experiment. (1) Temporal attention was manipulated via precues that instructed the observers regarding the relevance of each stimulus. (2) Temporal expectation was manipulated with the probabilities of when the targets may occur: (2a) Temporal precision was defined by the variability of the target onset within the experimental session; in low-precision sessions, (2b) Hazard rate was defined by the increasing probability of the target given it has not yet occurred until that moment. We measured visual performance for each combination of temporal attention and temporal expectation manipulations (Temporal attention x Temporal Precision, Temporal Attention x Hazard Rate), using discriminability index (d’), reaction time (RT) and balanced integration score (BIS) to combine discriminability with reaction time, and performed statistical tests on these measures.

### Statistical analysis

Discriminability index (d’), reaction time, and balanced integration score (BIS) were computed separately for each experimental condition (temporal attention and two forms of temporal expectation). Data analyses were performed with R (version 4.2.3; R Core Team, 2023), with ANOVA conducted using the ezANOVA package (version 4.4–0; Lawrence, 2016).

The discriminability index, d’ was computed by: z(hit rate) – z(false alarm rate). Correct responses in trials where the response-cued Gabor was oriented clockwise trials were categorized as hits and incorrect responses in trials where the response-cued Gabor was oriented counter-clockwise trials were categorized as false-alarms^34,36–39^. We implemented a correction to avoid infinite values when computing *d’*, and added 0.5 to the number of hits, misses, correct rejections, and false alarms before computing *d’*^40,41^.

We use median values for RTs (from the go-cue) throughout the analysis as the participants had infinite time to respond, causing the RT distribution to be skewed, which makes the median a less biased estimator for RT.

To capture the effects on accuracy and the speed of responses with a single metric, we also calculated the Balanced Integration Scores (BIS):

$${\text{BIS}}_{{{\text{i}},{\text{j}}}} = {\text{ ZACC}}_{{{\text{i}},{\text{j}}}} - {\text{ ZRT}}_{{{\text{i}},{\text{j}}}}$$

Here, BIS_i,j_ represents the difference between standardized mean correct accuracies and median RTs, affording equal importance to each. The subscripts, i and j, correspond to participant i's performance for condition j^42^.

## Results

We are primarily interested in whether temporal attention (valid performance with respect to neutral) varies with temporal expectation (precision and hazard rate). The SOA between the targets were fixed to 250 ms, which made T2 temporally predictable once T1 was presented, as T1 served as a temporal anchor to T2. In our analyses, we include “target” as a factor to account for this. The target-specific effects revealed in the analysis may be due to the availability of a temporal anchor, whereas the effects that occur similarly for both targets mean that these modulations occur early in the trial and are not modulated by the temporal anchor.

### Temporal precision

To investigate whether temporal attention interacts with temporal precision, we analyzed the data where the stimuli occurred at the expected time points (1400 ms for T1 and 1650 ms for T2 relative to the precue onset). In such trials, the trial timings and experimental manipulations were exactly the same, except that those trials were embedded in sessions with different temporal probabilities (Certain, Narrow, Wide, Uniform conditions), resulting in different levels of temporal precision.

We expected improvement in visual performance with temporal attention, such that performance in valid trials would be higher than in neutral trials, yielding attentional benefits. We were particularly interested in whether attentional benefits would vary across the levels of temporal precision. No interaction between precue (attention) and temporal precision (expectation) would indicate that temporal expectations do not guide temporal attention, and provide evidence for independence of these mechanisms. Alternatively, an interaction between precue and temporal precision due to a larger performance benefit for valid over neutral trials with increasing precision would indicate that temporal attention and expectation act together to modulate visual performance, and expectations are likely to guide attention.

We performed three-way ANOVAs (2 target × 2 precue × 4 precision) on sensitivity (d’), criterion, reaction time (RT), and Balanced Integration Scores (BIS). The factors analyzed were target (T1, T2), cue validity for temporal attention (Valid, Neutral), and temporal distribution for the temporal precision (Certain, Narrow, Wide, Uniform; in which the target occurred at the expected moments with a respective probability of 100%, 86%, 42%, and 33%). We registered temporal distribution as a numeric variable in order to account for the spacing between the probability values^43^.

**Figure 3:**
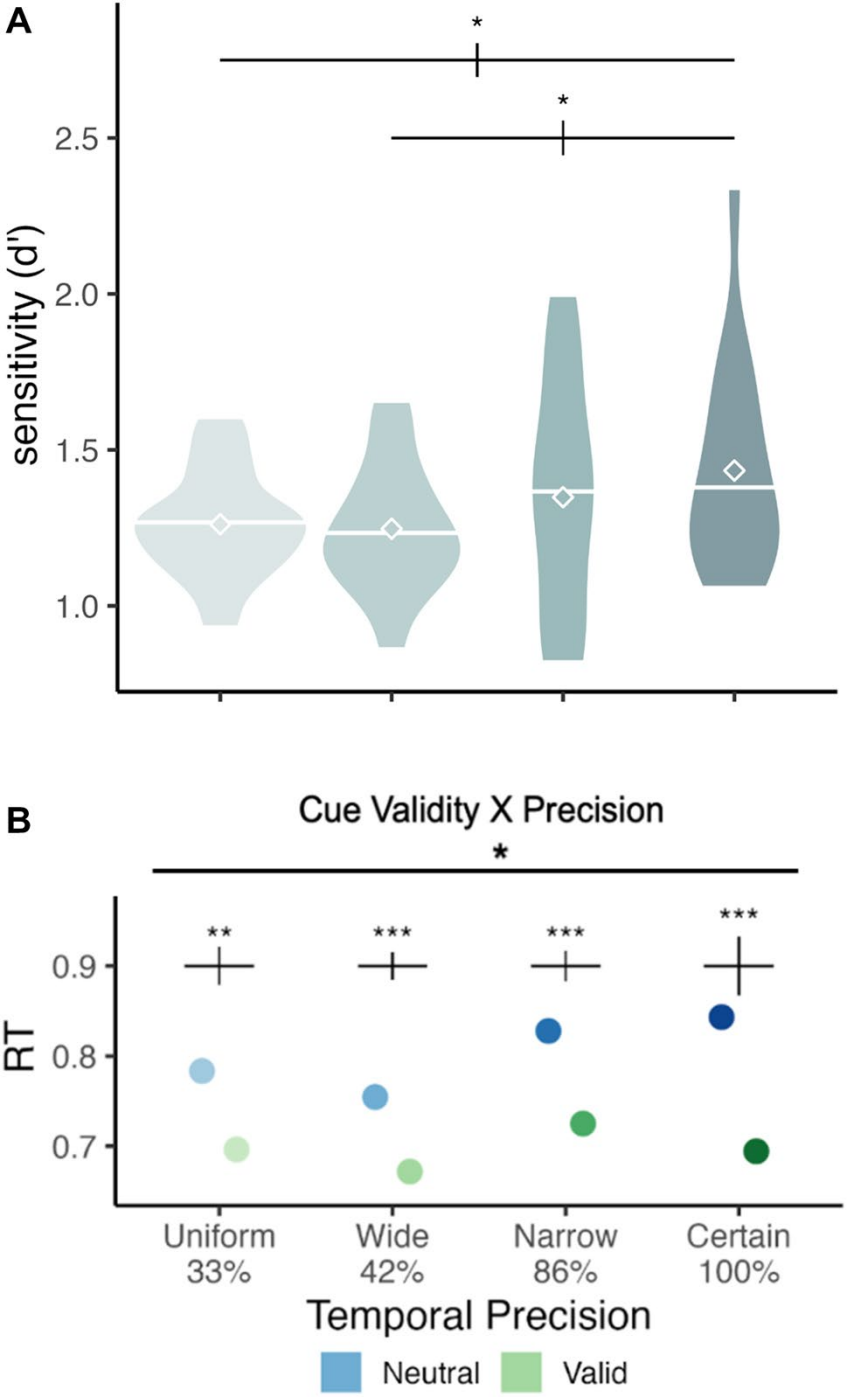
d’ and reaction time computed at the expected time point of all precision conditions. Only trials where T1 and T2 appeared at the expected time point (1400 and 1650, respectively) were included in the analysis. Error bars above each group of bars denote within-subject error rate with Morey correction^48,49^. Diamond icons and the horizontal bars overlaid on the violin plots represent mean and median values, respectively. (**A**) Main effect of temporal precision on discriminability (d’). The results suggest a gradual increase in overall performance with increasing precision. (**B**) The interaction between cue validity and temporal precision on reaction time. The benefit of temporal attention was present across the levels, although it gradually increased with precision.

**Figure 4:**
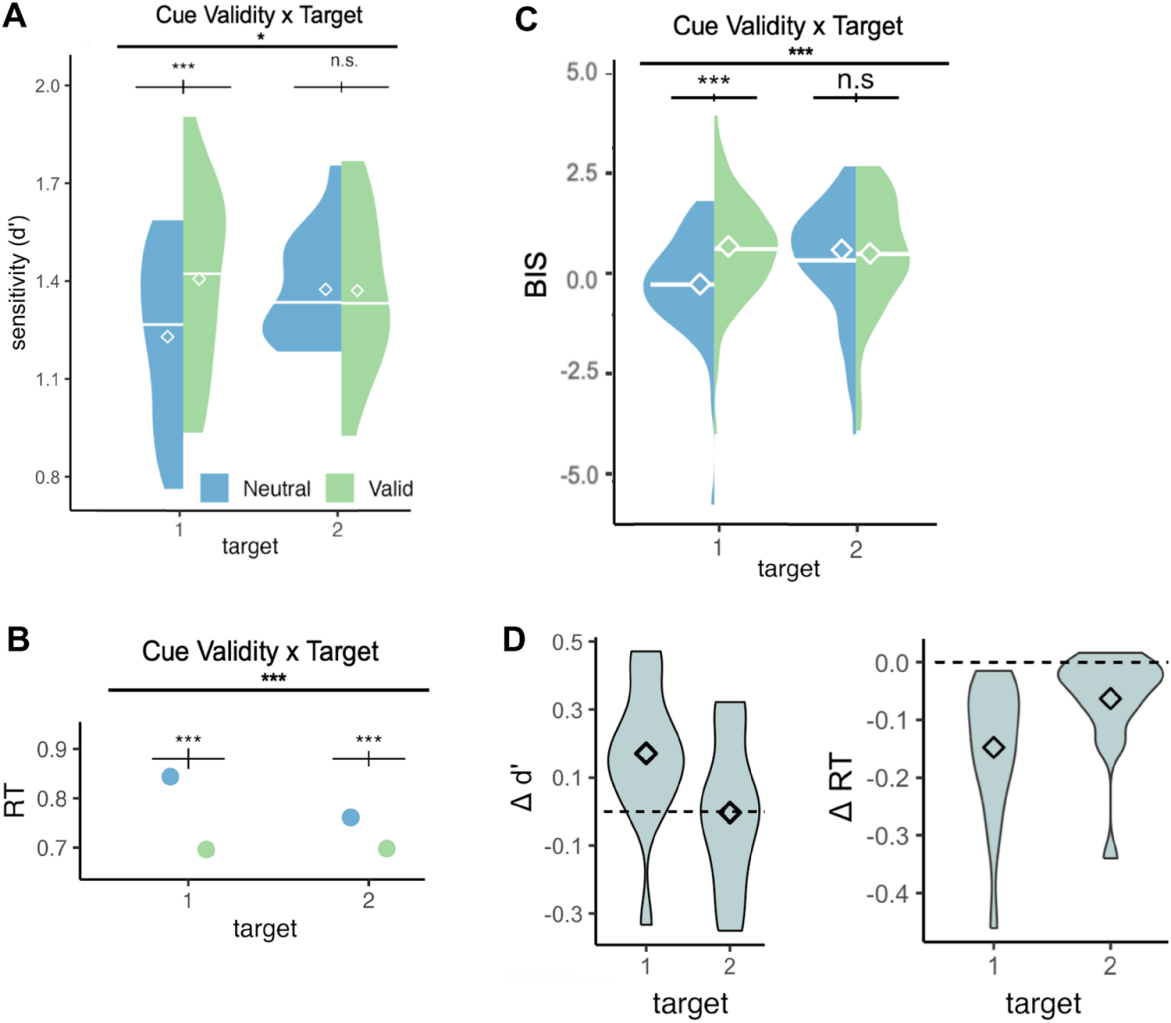
We analyzed visual performance with respect to temporal precision by focusing on the trials in which the targets appeared at the expected moment/middle point of each temporal distribution (T1 onset 1400 ms, and T2 onset 1650 ms), such that the stimulus timings were equivalent in those trials, and the only difference was the temporal distribution (precision) in which those trials were embedded. Diamond icons and the horizontal bars overlaid on the violin plots represent mean and median values, respectively. Significant interactions between cue validity and target were present for (**A**) discriminability (d’), (**B**) response time (RT) and (**C**) Balanced Integration Score (BIS) across temporal precision levels. Higher d’, BIS and faster RT emerged in the valid than neutral condition for T1, but there was either smaller (for RT) or no (for d’ and BIS) such difference for T2. (**D**) The differential attentional modulations were visualized by subtracting visual performance (d’ and RT) in valid trials from neutral trials. Dashed horizontal line marks 0 difference, and large deviation from 0 indicates attentional benefit.

Significant main effects for d’ were found for cue validity, F(1,15) = 5.180, p = 0.038, *η*_*G*_^2^ = 0.029, and temporal distribution (Fig. 3.A), F(1,15) = 9.341, p = 0.008, *η*_*G*_^2^ = 0.080. Furthermore, a significant interaction between target and cue validity was found, F(1,15) = 5.757, p = 0.030, *η*_*G*_^2^ = 0.031 (Fig. 4.A). Holm-corrected pairwise t-tests showed that d’ was higher than Valid than Neutral condition for T1, p = 0.0003, and there was no significant difference between those conditions for T2 (p = 0.520) (Fig. 4D). Pairwise t-tests for temporal distribution also showed that d’ for Certain was higher than d’ for Wide, p = 0.031, and that d’ for Certain was higher than d’ for Uniform, p = 0.021. Overall, accuracy increased with temporal attention for the first target, and across targets, it gradually increased with temporal precision.

The ANOVA on the criterion revealed no significant main effects or interactions (All ps > 0.1).

For RT, we found a significant main effect for cue validity, F(1,15) = 17.696, p = 0.001, *η*_*G*_^2^ = 0.231. Additionally, significant interactions were found between cue validity and temporal distribution, F(1,15) = 5.869, p = 0.029, *η*_*G*_^2^ = 0.014 (Fig. 3.B), and between target and cue validity, F(1,15) = 36.979, p < 0.0001, *η*_*G*_^2^ = 0.046 (Fig. 4.B). The holm-corrected pairwise comparisons revealed faster RTs in valid than neutral conditions (p < 0.0001), and although this was present for both targets, it was more pronounced for T1 than T2 (p < 0.0001 for T1 and p = 0.0001 for T2) (Fig. 4D). And the same effect, reduction in RT with attention was observed in all temporal precision conditions, even though it was the smallest under lowest precision (p = 0.0004 for uniform (33%), p = 0.0001 for wide (42%), and p < 0.0001 for other levels). In sum, temporal attention facilitated reaction times, and more so for the first target, and this attentional modulation increased with temporal precision.

**Figure 5:**
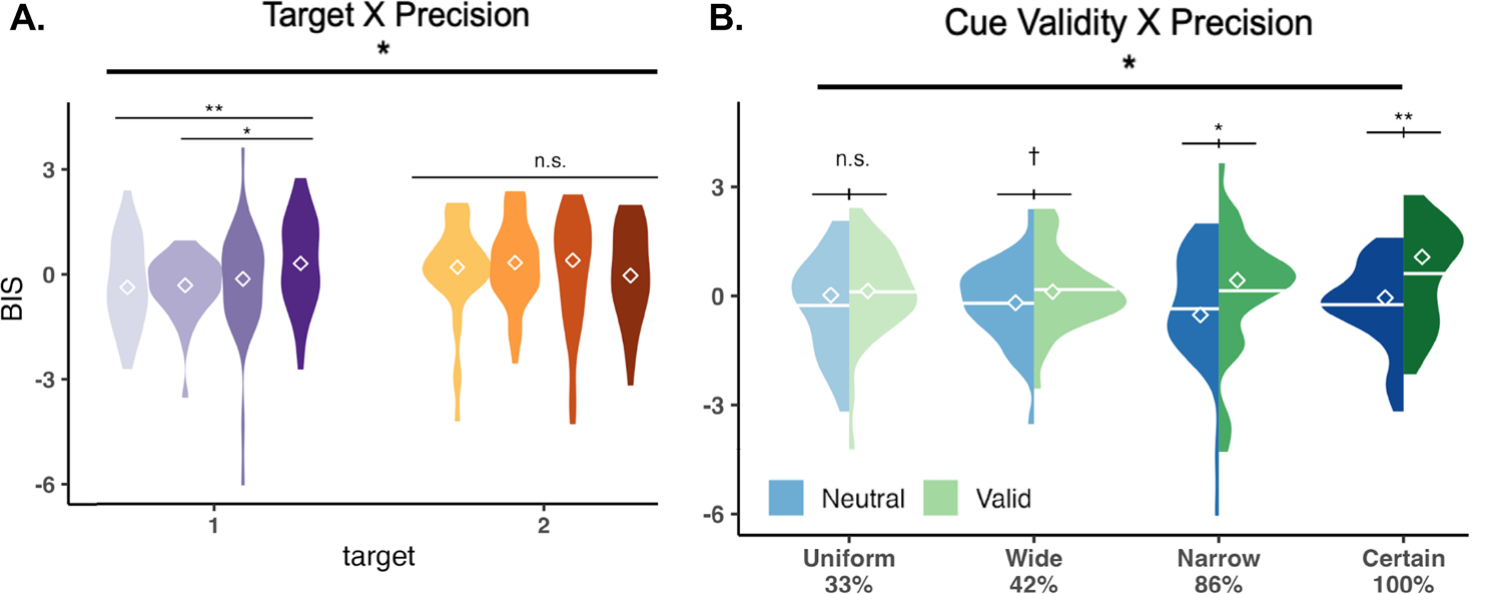
Trials in which the targets appeared at the expected moment/ middle point of the temporal distributions (T1 onset 1400 ms, and T2 onset 1650 ms) were analyzed, such that the stimulus timings in these trials were the same, but the temporal precision was variable. Temporal precision is indicated with the darkness of the colors in the figures. (**A**) There was a significant interaction between target and temporal precision. The level of temporal precision (temporal variability of the experimental session) affected T1, but not T2 at the same expected moment. (**B**) There was a significant interaction between temporal precision and cue validity. Temporal attention benefits performance when target timing is precisely predictable in narrow and certain conditions, and the magnitude of benefit increases with precision.

For BIS, a significant main effect was found for cue validity, F(1,15) = 16.857, p = 0.001, *η*_*G*_^2^ = 0.137. Moreover, significant interactions were found between target and cue validity, F(1,15) = 23.724, p = 0.0002, *η*_*G*_^2^ = 0.072 (Fig. 4C), between target and precision, F(1,15) = 5.134, p = 0.039, *η*_*G*_^2^ = 0.039 (Fig. 5A), and between cue validity and precision, F(1,15) = 6.095, p = 0.026, *η*_*G*_^2^ = 0.017 (Fig. 5B). Holm-corrected pairwise t-tests between cue validity and target revealed a higher BIS score for the T1 Valid than the Neutral condition, p < 0.0001, and no effect for the T2, p = 0.170. Pairwise t-tests for cue validity and temporal distribution showed a significantly higher BIS score for the Certain Valid than the Neutral, p = 0.0001, for the Narrow Valid than the Neutral condition, p = 0.004, and a statistical trend for the Wide Valid than the Neutral, whereas no effect was found for the uniform condition. Pairwise comparisons revealed that for T1, observers had a significantly higher performance with certain timing (100%) than uniform (p = 0.004) and wide temporal distributions (p = 0.013). For T2, there was no significant difference across the levels of temporal precision. To summarize, visual performance was improved by temporal attention and temporal precision for the first target. Moreover, across the targets, temporal attention and precision interacted, and the benefits of temporal attention increased with temporal precision.

### Hazard rate

Hazard rate is another form of temporal structure that our brain uses to form temporal expectations^15,21^. Unlike temporal precision, which is based on the temporal variability of the context or experimental session and cannot be inferred from a single event or trial, hazard rate can be instantaneously computed within each trial. In our experiment, two targets appeared in all of the trials and the possible time windows for the target onsets were constrained. Therefore, as time passed and the targets had not appeared in a given trial, the probability of the targets’ appearance in the next moment increased. This relation is characterized by a monotonic increasing hazard function that describes hazard as a function of time (hazard rate). In this experiment we used symmetric temporal distributions that peaked at the midpoint, which allowed us to describe the target onsets as “earlier”, “at”, or “later” than the expected moments. Therefore, although the temporal probabilities for the early and late stimulus onsets are equal, they differ in terms of hazard rate, such that it is lower for the early stimulus onsets and higher for the late onsets.

To analyze visual performance modulations with temporal attention and hazard rate, we analyzed the entire time window in the wide (42%) and uniform (33%) conditions, as these two conditions had enough trials at the unexpected time points. We collapsed the data across these two conditions. We categorized the stimulus onsets relative to the expected moment: Early, Expected, Late onsets.

Overall, we predicted that performance would be higher in valid than neutral trials–attentional benefits. If temporal attention were sustained or quickly reoriented, results would show equal benefits for valid over neutral trials across time regardless of the stimulus onset, yielding no interaction between precue and stimulus onset. However, a significant interaction between attention and expectation could emerge in two alternative ways. If temporal attention was not sustained and expectation guided attention, the largest attentional benefits would happen at the expected moment, and smaller or no attentional benefits earlier or later than expected. Alternatively, temporal attention benefits may vary with hazard rate. Lower attentional benefits in the later than earlier window would indicate suboptimal allocation of temporal attention, and no evidence of attentional reorienting.

The potential interaction between temporal expectation and attention on d’, criterion, reaction time (RT), and balanced integration scores (BIS) was assessed by three-way ANOVAs (2 × 2 × 3). The factors were target (T1, T2), cue validity (Valid, Neutral), and the stimulus onset (Early, Expected, Late). For this analysis, we did not register stimulus onset as numerical, as the independent variables were evenly spaced (but results were the same when we did so).

**Figure 6:**
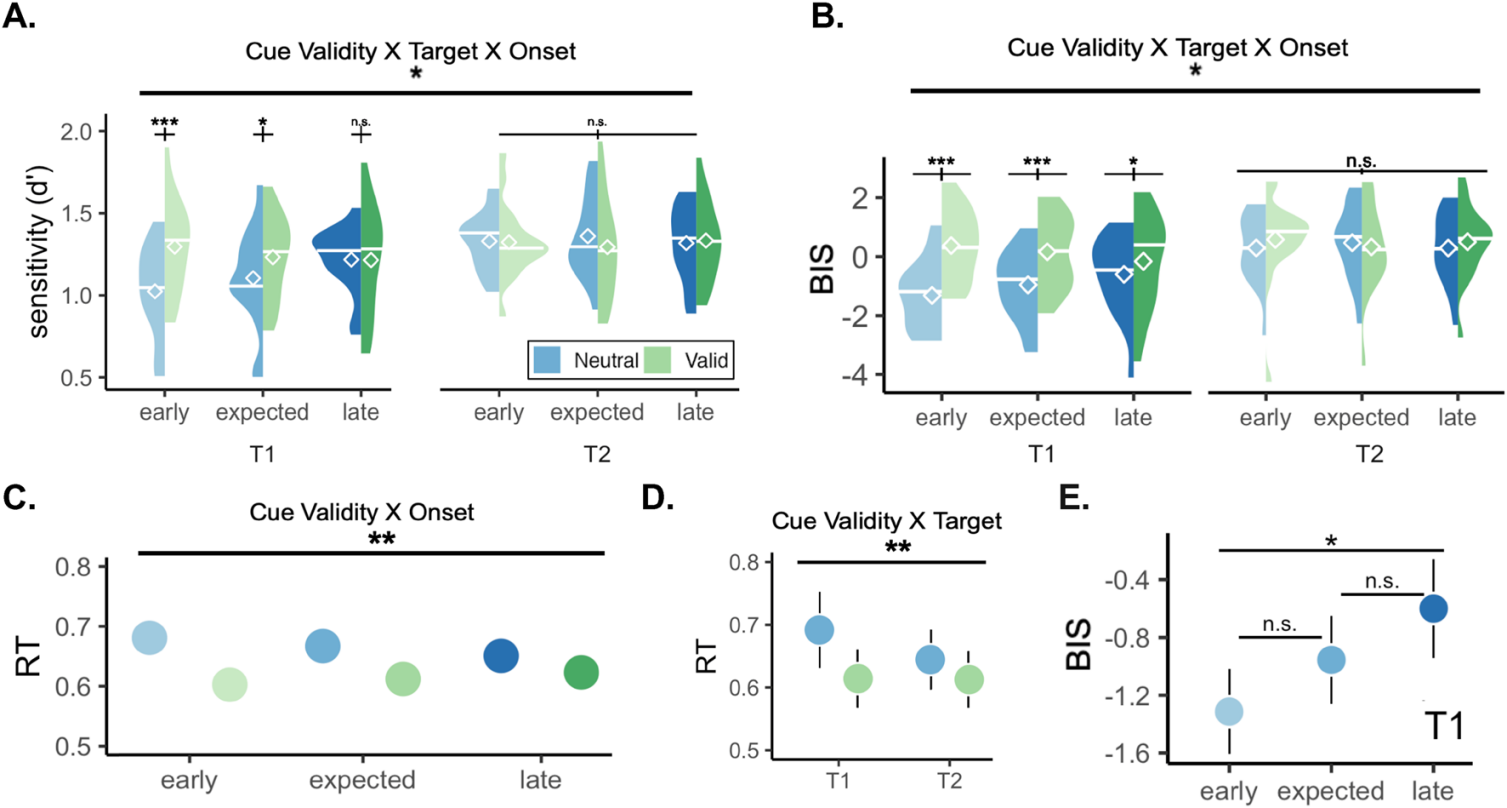
Performance was analyzed across the time window that the stimuli could appear (wide 42% and uniform 33% temporal precisions). (**A**,**B**) Analyses on d’ and BIS revealed three-way significant interactions between target, stimulus onset and cue validity. Temporal attention improved performance for T1, but not T2. The improvements of temporal attention (the difference between valid and neutral at each time point) for T1 were absent after the expected moment. (**C**) Benefits on RT were present across the time window (all ps < 0.001), although this effect got smaller as the stimulus onset was delayed (revealed by an interaction between the stimulus onset and cue validity). (**D**) Temporal attention benefits on RT were present for both targets (both ps < 0.001), although was stronger for T1 than it was for T2, indicated by a significant interaction between target and cue validity. (**E**) Post-hoc analysis on T1 performance in neutral trials. Same data from (**B**) replotted to show the statistical findings.

The analysis on d’ revealed significant interactions between target and cue validity, F(1,15) = 9.705, p = 0.007, *η*_*G*_^2^ = 0.021, and between cue validity and stimulus onset, F(2,30) = 4.656, p = 0.017, *η*_*G*_^2^ = 0.011. A significant three-way interaction between target, cue validity, and stimulus onset was also present, F(2,30) = 3.537, p = 0.042, *η*_*G*_^2^ = 0.015 (Fig. 6.A). No significant main effect for the stimulus onset (p > 0.1). A statistical trend for target was found (p = 0.076). Holm-corrected pairwise t-tests for the three-way interaction revealed a significant difference between Neutral and Valid conditions for T1 at early onsets (p = 0.0001), followed by a smaller effect at the expected onset (p = 0.034), and there was no effect of temporal attention at late onsets (p = 0.52). No significant effect of cue validity was found for T2 across the time window. Temporal attention improved discriminability for the first target, specifically when the target appeared earlier than, or at the expected moment, but not later.

A three-way ANOVA on the criterion did not reveal any significant main effect or interaction (All ps > 0.1).

With regard to RT, a significant main effect was observed for cue validity, F(1,15) = 17.670, p = 0.0007, *η*_*G*_^2^ = 0.041, and for target F(1,15) = 6.969, p = 0.018, *η*_*G*_^2^ = 0.025. Significant interactions were found between target and cue validity, F(1,15) = 16.195, p = 0.001, *η*_*G*_^2^ = 0.008 (Fig. 6.D), and between cue validity and stimulus onset, F(2,30) = 7.090, p = 0.003, *η*_*G*_^2^ = 0.0008 (Fig. 6.B). Pairwise t-tests between stimulus onset, cue validity, and target revealed lower RT in valid than neutral conditions for both T1 and T2 across the entire temporal window (all ps < 0.001), but this effect was stronger for T1 than T2, and stronger at early, than expected, followed by the late onsets. Temporal attention facilitated RT, and this facilitation was the strongest for the early targets for T1.

For BIS, significant main effects were found for target, F(1,15) = 7.803, p = 0.014, *η*_*G*_^2^ = 0.093, and cue validity, F(1,15) = 7.351, p = 0.016, *η*_*G*_^2^ = 0.051. There were also significant interactions between target and cue validity, F(1,15) = 26.289, p = 0.0001, *η*_*G*_^2^ = 0.034 (Fig. 6D), and between cue validity and stimulus onset, F(2,30) = 8.085, p = 0.002, *η*_*G*_^2^ = 0.012, and a three-way interaction between target, cue validity, and stimulus onset, F(2,30) = 4.546, p = 0.018, *η*_*G*_^2^ = 0.01 (Fig. 6C). Pairwise comparison between cue validity for the target at each time point showed a higher BIS score for the T1 Valid than Neutral condition that got smaller with stimulus onset, with the strongest difference at early onsets (p < 0.0001), followed by the expected moment (p = 0.0004), and followed by a statistical trend at late onsets (p = 0.085). No significant difference was found for T2 across the time window (all ps > 0.1). Overall, we found that temporal attention improved performance for T1, the strongest in the early time window, followed by the expected moment, and then with a minimal improvement when targets appeared later than expected.

We performed post hoc analysis on the neutral performance across time, using the BIS scores. Holm-corrected pairwise t-tests revealed a significant difference in T1 neutral performance at early and late time windows (p = 0.020), and no significant difference between early-expected, or late-expected windows (both ps > 0.1) (Fig. 6E). We describe this post-hoc finding as “temporal expectation benefit”, as the baseline (neutral) performance increased with hazard rate.

## Discussion

This study revealed an interaction between voluntary temporal attention and temporal expectation. The benefits of temporal attention on performance depended on expectation: They gradually increased as a function of temporal precision (Fig. 5B); conversely, and surprisingly given previous findings, they decreased with the onset delay of the behaviorally relevant stimulus (Fig. 6), despite its increasing probability of appearance.

### People use expectation suboptimally to guide attention to a specific point in time

Relevant moments can be selected and prioritized through endogenous temporal attention when events appear at certain time points, resulting in perceptual benefits and costs at attended and unattended times, respectively^3,20,32^. The protocols in these studies isolated temporal attention and expectation by keeping the timing of the sequential stimuli constant. Thus, whether expectation needed to be temporarily precise, or is even necessary for temporal attention allocation was an open question. Perceptual tradeoffs in these experiments could have been attained merely by selecting a visual event based on the order in which they appear in a sequence, regardless of temporal expectation (e.g., the first event among the sequence), or by considering temporal expectation, by estimating the probabilities of when events would occur, and directing attention to a specific time point beforehand (e.g., 1 s after the precue). The protocol we used here enabled us to disentangle these alternatives. According to the former possibility, temporal attention would be sustained and the results would reflect uniform benefits across the tested time window. According to the latter possibility, temporal attention should peak at the expected moment, and would gradually increase as a function of precision of the expectation. Our results revealed that temporal precision modulated attentional benefits on visual performance, assessed by Balanced Integration Score that combined discriminability and reaction time (Fig. 5B), hence expectation needed to be temporally precise. Moreover, temporal attention benefits on visual performance (assessed by d’, RT and BIS, Fig. 6A-C) were not constant over time across the hazard function, which describes hazard as a function of time (the conditional probability of the stimulus occurring at a specific time point, given that it has not occurred yet). This finding indicates that attentional allocation goes beyond the stimulus order in the sequence. Interestingly, temporal attention benefit was higher for earlier than the expected time points and gradually decreased with onset delay for RT and BIS, and disappeared at the late window for d’. These results indicate that temporal attention cannot be sustained across time despite constant competition between two sequential stimuli, and suggest that it should be precisely allocated.

We interpret the higher benefit of temporal attention before the expected time to reflect suboptimal allocation. Optimal allocation of temporal attention would have yielded the highest benefits at the expected moment, and symmetrical benefits at earlier and later moments. Instead, we found an *attentional undershoot–*the highest benefits at early moments, followed by the expected moment, and minimal benefit at the later window. In the current design the late stimulus onset could not be predicted at the beginning of the trial, and if attentional resources were spent they could not be reallocated. In contrast, temporal attention can benefit performance at the same late time window when there is no temporal uncertainty and resources could be saved to be deployed at the appropriate time^20^.

### Temporal attention benefits vary as a function of temporal precision

The dynamic normalization model of temporal attention explains the temporal attention effects when stimulus timing is certain, and assumes precise selection of those behaviorally time points^3^. It has been pointed out that this model does not differentiate whether the behavioral modulations are attained through differential anticipation before the two stimuli, and/or differential prioritization and filtering of the relevant stimulus during (or after) sensory encoding^44^. Our new protocol and findings address this point. They reveal that temporal attention is not sustained across time, and a gradual decrease in benefits on d’, RT and BIS with a delayed stimulus onset suggests that temporal attentional selection occurs prior to the stimulus onset. We also highlight that a readout effect after the sensory encoding would result in the same performance for valid and neutral conditions in the prior^3,20,32^ and the present studies.

Temporal expectation has been shown to have different sources in the auditory domain. In a study that used a similar temporal expectation manipulation as we did, the neural response amplitudes decreased when expectations increased, regardless of whether the auditory event onset was delayed within trials, or when the stimulus onset variability was smaller. However, the temporal expectation developed through passage of time within the trial evoked earlier responses in early auditory regions and superior temporal gyri, whereas expectations formed via temporal variability result in delayed responses in the parietal cortex^30^.

In the current study, we used a similar protocol to manipulate expectations, so that temporal expectations increased as a function of both temporal precision (within-session temporal variability; the less the variability, the higher the expectation precision) and hazard rate (within-trial increasing expectations and conditional probability of the target onset as the onset is delayed). We found that the benefits of temporal attention interact with expectation, but the direction of the modulation is opposite for the two sources. On the one hand, with increasing expectations, based on temporal precision, attentional benefits on d’, RT and BIS gradually increased. On the other hand, temporal attention benefits decreased as the stimulus onsets were delayed via the hazard rate. Our behavioral findings provide further evidence for the hypothesized distinction between the two sources of temporal expectation in the auditory domain.

Previous studies concluding that expectations can guide temporal attention report a temporal asymmetry when there is a mismatch between the participants’ expectation and the target onset; lower performance when a target appears earlier than at an expected moment than when it appears later than it. The authors interpret the findings as expectation guidance of attention to an early time point in the trial, and that when the cue is invalid, hazard rate guides observers to voluntarily reorient attentional resources to a later time point^21–26^. However, these two processes were not independently manipulated in their experiments.

Our study distinguishes between temporal attention and expectation, and between two sources of expectation: we found that expectations that are known beforehand–temporal precision–do indeed modulate temporal attention. Furthermore, our results revealed that temporal attention cannot be reoriented with evolving probabilities when the target onset is delayed. Had temporal attention been flexibly reoriented as guided by the hazard rate, we would have observed temporal attention benefits on d’, RT and BIS at the late time window.

Thus, our results suggest that the above-mentioned previous behavioral improvements could be explained by temporal expectations, without invoking the allocation of temporal attention. This idea is also supported by our finding that baseline performance in the neutral condition increased with hazard rate, which we described as “expectation benefit”. We note that the lack or decrease of attentional benefit at late intervals in our study could be due to the shorter temporal window (400 ms) than in the designs of the aforementioned studies (500–1000 ms). According to the dynamic normalization model of temporal attention, the estimated recovery time of voluntary attention was 918 ms^3^). But the recovery time of temporal attention still needs to be systematically evaluated.

Exogenous temporal attention–involuntary selection of specific time points following brief salient changes–interacts with expectation. It improves performance when the stimuli are presented at a later time point, but not earlier, in the trials in which stimuli onset probability is uniform^4^. The current findings show that endogenous, voluntary temporal attention interacts with expectations in an opposite manner; they were pronounced at the early window and minimal at the late window. These opposite findings indicate a distinction between exogenous and endogenous attention, akin to the well-established distinction of these subsystems in spatial attention^34,36,45–47^

In conclusion, this study highlights that endogenous temporal attention and expectation are distinct mechanisms whose effects depend on contextual and goal demands. Temporal expectation can guide attention; the more precise the timing the higher the benefits; however, endogenous attention is suboptimally allocated at earlier than expected moments, and once allocated, it cannot immediately be reoriented to a later time point if visual events are delayed.

## Data Availability

The data and codes for the study are available upon request from the corresponding author, Aysun Duyar.
